# Real-Time SpO_2_ Estimation from Chest Reflectance Photoplethysmography: Algorithm Development and Clinical Validation Against Arterial SaO_2_

**DOI:** 10.3390/s26175676

**Published:** 2026-09-07

**Authors:** WooYong Lee, Jaeyeon Shin, MiHye Song, Mi-Kyung Song, Kyung Mi Kim, Gyu-Jeong Noh, SungPil Cho

**Affiliations:** 1 Department of Development, MEZOO Co., Ltd., 200, Gieopdosi-ro, Jijeong-myeon, Wonju-si 26354, Republic of Korea; wylee@me-zoo.com (W.L.);; 2 Department of Anesthesiology and Pain Medicine, Asan Medical Center, University of Ulsan College of Medicine, Seoul 05505, Republic of Korea; 3 Department of Clinical Pharmacology and Therapeutics, Asan Medical Center, University of Ulsan College of Medicine, Seoul 05505, Republic of Korea

**Keywords:** PPG, SpO_2_, SQI, chest patch, reflectance oximetry, wearable device

## Abstract

Peripheral oxygen saturation (SpO_2_) estimation using photoplethysmography (PPG) is typically based on the pulsatile components of red and infrared (IR) PPG signals acquired from peripheral sites, such as the finger or earlobe. Although these sites provide strong PPG signals, they are less suitable for integrated monitoring with electrocardiography (ECG), including arrhythmia detection. Simultaneous acquisition of PPG and ECG from a chest-worn device may therefore enable continuous and integrated physiological monitoring. However, chest PPG is characterized by lower perfusion and greater susceptibility to respiratory and motion artifacts, making reliable extraction of pulsatile red and IR components challenging. In this study, we developed and clinically evaluated a real-time SpO_2_ estimation algorithm based on chest reflectance PPG. Green PPG, which provides a relatively distinct pulsatile waveform under low-perfusion conditions, was used for beat detection. A heart-rate-adaptive analysis window, normalized cross-correlation-based signal quality assessment, and least-squares estimation of the AC amplitude ratio were combined to calculate the red-to-IR ratio of ratios, from which SpO_2_ was estimated using a calibration equation. Algorithm performance was evaluated against reference arterial oxygen saturation (SaO_2_) measured using a blood gas analyzer. The proposed method achieved an accuracy root mean square of 2.91% relative to the reference SaO_2_. These results support the technical feasibility of chest reflectance PPG-based SpO_2_ estimation and its accuracy under controlled desaturation conditions.

## 1. Introduction

Recent advances in wearable technology have focused on the simultaneous measurement of multiple physiological parameters, including heart rate, respiration, oxygen saturation, and electrocardiogram (ECG), using a single wearable device [1,2]. Integrating multiple physiological measurements into a single device can reduce the burden of sensor attachment and user discomfort compared with measuring each parameter separately, while providing complementary physiological information. Therefore, such technologies have substantial potential for applications in daily health management, remote patient monitoring, and long-term continuous monitoring.

Among these physiological parameters, oxygen saturation is particularly relevant to continuous cardiopulmonary monitoring because pulse oximetry provides a noninvasive estimate of arterial oxygen saturation using photoplethysmography (PPG). PPG-based pulse oximetry is well suited to wearable implementation because of its noninvasive nature, compatibility with compact optical sensors, and ability to be integrated with other physiological measurements [3]. In particular, incorporating SpO_2_ estimation into a chest-worn platform that already acquires ECG and other cardiorespiratory signals may enable multimodal monitoring from a single anatomical site and reduce reliance on additional peripheral sensors.

Previous studies on PPG-based oxygen saturation measurement and commercial pulse oximeters have primarily focused on peripheral sites, such as the finger, earlobe, and wrist, where PPG signals can be acquired with relatively large amplitudes and high stability [4]. At these sites, peripheral oxygen saturation (SpO_2_) is conventionally estimated by calculating the ratio of ratios (*R*) from the pulsatile (AC) and direct-current (DC) components of red and IR PPG signals and subsequently applying an empirically derived calibration equation [4,5]. Unlike transmission-mode pulse oximetry, which requires light to pass through the tissue, reflectance-mode pulse oximetry allows the light source and photodetector to be positioned on the same surface. This configuration enables measurements at various anatomical sites, including the foot, forehead, wrist, finger-ring interface, and chest [6,7]. Accordingly, with the growing demand for wearable sensors, the range of applications for reflectance PPG-based oxygen saturation measurement has continued to expand. Wrist-worn reflectance pulse oximeters have demonstrated utility for monitoring oxygen saturation during sleep and assessing sleep apnea [8]. In addition, ring-type devices have shown the potential for high-sensitivity and low-power measurements through optimization of their optical configurations [9]. These developments have stimulated active research into reflectance-mode wearable devices with diverse form factors. Recent studies have also highlighted the chest as a promising site for multimodal physiological monitoring. Long et al. demonstrated photonic sensing of seismocardiographic signals [10], and Qiu et al. developed a multimodal chest patch for simultaneous ECG, heart-sound, and respiratory monitoring [11]. These studies further support the potential of chest-worn platforms for integrated cardiorespiratory monitoring.

However, peripheral-site measurements have limitations for long-term, continuous, and unobtrusive monitoring. In cold environments or under conditions of impaired peripheral circulation, reduced PPG signal amplitude may compromise measurement reliability [12]. In particular, finger-based oxygen saturation monitoring may restrict user activity during prolonged wear, while motion during daily activities may degrade signal quality [13]. In contrast, the chest is suitable for long-term monitoring using a wearable patch and has a relatively limited effect on user activity [14]. Moreover, because ECG and PPG signals can be acquired simultaneously from the chest using a single device, the chest may serve as an advantageous measurement site for the integrated monitoring of oxygen saturation and ECG [15].

Nevertheless, important technical challenges remain in estimating SpO_2_ using chest reflectance PPG. Compared with peripheral sites such as the finger and earlobe, the chest generally exhibits lower perfusion, resulting in smaller pulsatile components in the red and IR PPG signals. Chest PPG is also highly susceptible to motion artifacts caused by respiration and body movement. Consequently, stable estimation of the AC components using conventional peak- and valley-detection methods is difficult, which may reduce the accuracy of the *R* calculation and subsequent SpO_2_ estimation [15,16,17]. In particular, a previous study [15] demonstrated the feasibility of sternum-based reflectance pulse oximetry; however, that study primarily focused on offline analysis and feature extraction. Real-time motion artifact detection, signal quality assessment, and stabilization of SpO_2_ output, which are essential for practical wearable devices, were not sufficiently addressed.

In this study, we developed a real-time SpO_2_ estimation algorithm based on reflectance PPG signals acquired using a chest-worn device (HiCardi M350; MEZOO Co., Ltd., Wonju-si, Gangwon-do, Republic of Korea) and implemented the algorithm in the M350 device. We also conducted a controlled desaturation study involving human participants and evaluated the accuracy of the proposed algorithm by comparison with reference SaO_2_ values obtained using an arterial blood gas analyzer. The study was designed in accordance with ISO 80601-2-61:2017, which was applicable at the time of study design, and with reference to FDA guidance for pulse oximeters. A prespecified accuracy root mean square ($A_{\mathrm{rms}}$) criterion of 4.0% or less was used for interpretation of algorithm accuracy under the controlled study conditions. To compensate for the instability of red and IR peak and valley detection caused by low chest perfusion and respiratory and motion artifacts, the proposed algorithm integrates green PPG-based beat detection, a heart-rate-adaptive analysis window, a normalized cross-correlation-based signal quality index, least-squares-based AC component estimation, and real-time post-processing. In contrast to previous sternum-based reflectance PPG studies that primarily focused on offline analysis and the feasibility of feature extraction, the present study implemented a complete real-time SpO_2_ estimation pipeline for use in a practical wearable device.

## 2. Methods

### 2.1. Study Design

This study was conducted as a clinical investigation to develop a real-time method for estimating SpO_2_ from a chest-worn reflectance PPG device and to evaluate its accuracy. The clinical investigation was designed as a controlled desaturation study in accordance with ISO 80601-2-61:2017 and consisted of two stages with non-overlapping participant cohorts. Stage 1 involved five healthy adult volunteers and was used for algorithm development and finalization of the calibration model. The final calibration model was locked before Stage 2 and was not modified using Stage 2 data. Stage 2 involved 12 healthy adult volunteers and was used for pivotal accuracy evaluation. In total, 17 participants were enrolled across the two stages, and the measurement procedures were identical. Because both stages were conducted within the same overall clinical investigation, the Stage 2 evaluation should be regarded as an independent within-study accuracy evaluation rather than as external validation.

### 2.2. Measurement Devices

**Figure 1:**
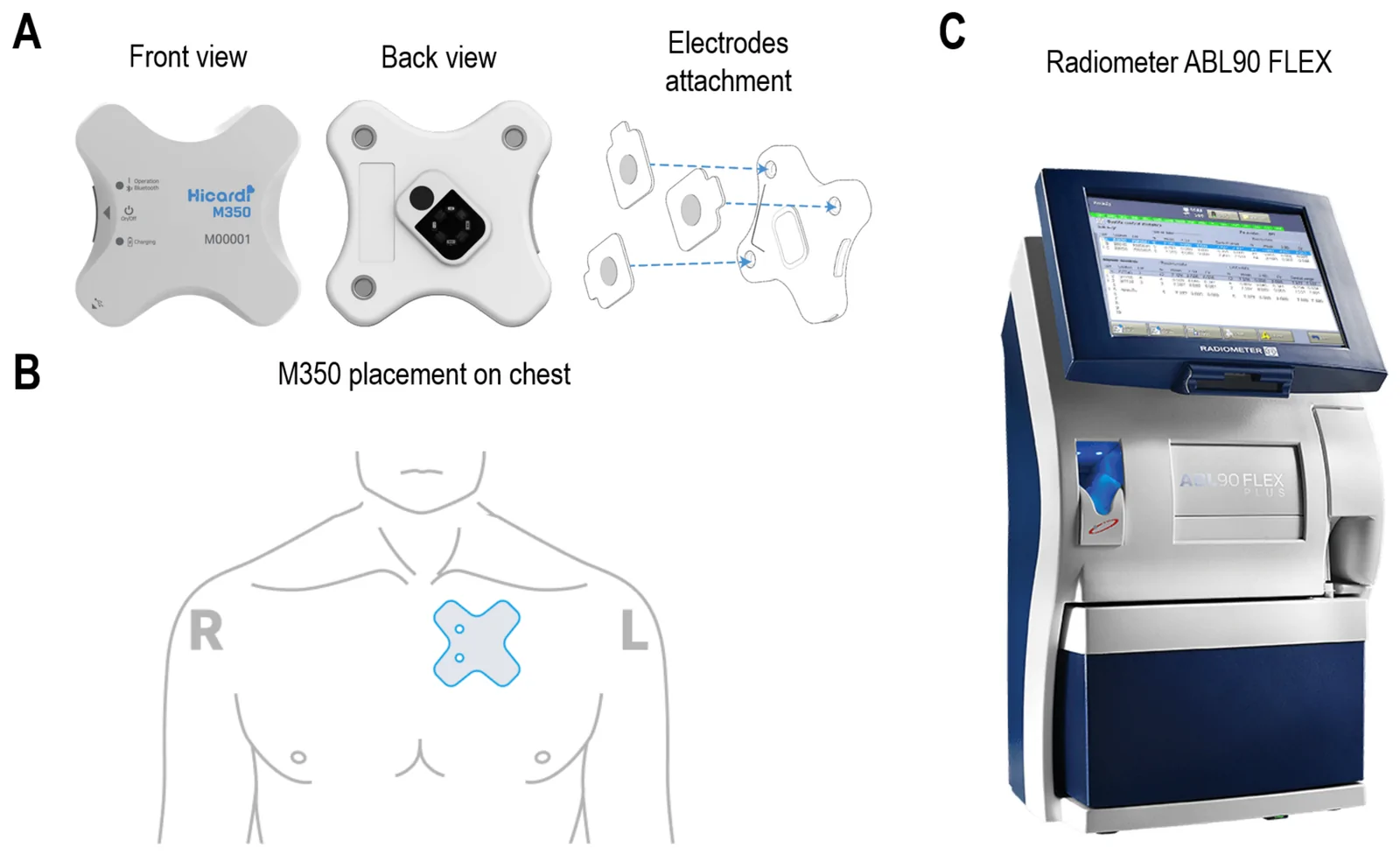
Measurement device and sensor placement. (**A**) Front and back views of the HiCardi M350 device and electrode attachment configuration. (**B**) Placement of the HiCardi M350 on the left anterior chest according to the left shoulder line. (**C**) Radiometer ABL90 FLEX PLUS blood gas and CO-oximetry analyzer used to determine the reference arterial oxygen saturation (model 393-092; Radiometer Medical ApS, Brønshøj, Denmark).

The HiCardi M350 (HiCardi M350; MEZOO Co., Ltd., Wonju-si, Gangwon-do, Republic of Korea) used in this study is a chest-worn patch-type patient monitoring device designed to simultaneously acquire green, red, and IR PPG signals, ECG, respiration, and accelerometer signals. After the electrodes had been attached, the device was affixed to the chest, as illustrated in Figure 1A. The sensor was positioned on the left anterior chest in alignment with the participant’s left shoulder line and secured to ensure stable contact of the electrodes and optical sensor with the skin (Figure 1B). The M350 acquires reflectance-mode PPG signals, with the red and IR PPG signals used for SpO_2_ estimation in this study. The green PPG, ECG, and accelerometer signals were used as auxiliary signals for beat detection, signal quality assessment, and motion artifact detection, respectively.

The SpO_2_ processing algorithm was implemented directly on the M350 using an nRF52832 microcontroller (Nordic Semiconductor ASA, Trondheim, Norway) with a 64-MHz ARM Cortex-M4F processor. At the PPG sampling rate of 125 Hz, corresponding to an 8-ms sampling interval, the measured worst-case execution time during SpO_2_ processing was approximately 1.6 ms, corresponding to 20.0% of the available sampling interval and leaving a timing margin of approximately 6.4 ms. The compiled firmware including the SpO_2_ processing functionality occupied 140.47 kB of flash memory and 52.33 kB of RAM, with incremental SpO_2_-related memory requirements of approximately 45.37 kB of flash memory and 22.80 kB of RAM. These measurements indicate that the SpO_2_ processing can be executed within the timing and memory constraints of the M350 embedded hardware.

Reference oxygen saturation was defined as SaO_2_ measured from arterial blood using an ABL90 FLEX PLUS blood gas and CO-oximetry analyzer (model 393-092; Radiometer Medical ApS, Brønshøj, Denmark), as shown in Figure 1C.

### 2.3. Participants

The final analysis included 12 healthy adult volunteers, comprising six men and six women. The mean age was 28.9±4.5 years, the mean height was 171.7±9.8cm, and the mean body weight was 70.0±11.6kg. Before participation, all participants received a detailed explanation of the study objectives, measurement procedures, anticipated risks, and criteria for study termination, and provided written informed consent. The study was conducted in accordance with procedures approved by the Institutional Review Board (IRB No. 2025-1580). The inclusion criteria were as follows: men and women aged 20 to 49 years; carboxyhemoglobin <3%; methemoglobin <2%; total hemoglobin concentration >10g/dL; voluntary agreement to participate in the clinical study with written informed consent; and the ability to understand the study instructions and participate throughout the entire study period.

### 2.4. Controlled Desaturation Protocol

To induce controlled hypoxemia, each participant’s nostrils were occluded with a nose clip, and the participant breathed through a mouthpiece connected to an open breathing circuit. A gas mixture consisting of medical air, N_2_, and CO_2_ was administered, and the inspired oxygen concentration was progressively reduced by adjusting the flow rates of nitrogen and medical air. CO_2_ was included to minimize respiratory alkalosis caused by hyperventilation during hypoxemia induction. Airway gases were continuously analyzed using a mass spectrometer, and the gas flow rates were adjusted to achieve and maintain each target oxygen saturation plateau.

A Nellcor OxiMax N-560 pulse oximeter sensor (N-560; Covidien LLC, Mansfield, MA, USA) was attached to the participant’s ring finger to monitor attainment and stability of the target plateaus in real time. The N-560 SpO_2_ measurements were used solely to guide adjustment of the gas mixture and were not used as reference values for the accuracy analysis. Reference oxygen saturation was defined exclusively as arterial SaO_2_ measured using the ABL90 FLEX PLUS blood gas and CO-oximetry analyzer (Figure 1C). Before arterial catheterization, Allen’s test was performed to confirm adequate collateral circulation. Collateral circulation was considered adequate when normal palm color returned within 10 s after release of ulnar artery compression. Following local anesthesia with 0.5 mL of lidocaine, a 22-gauge catheter was inserted into the radial artery for serial arterial blood sampling.

Hypoxemia was induced stepwise over an SpO_2_ range of 70–100%, with target plateaus of 94, 90, 85, 80, 75, and 70%. The desaturation procedure was performed twice, with 100% oxygen administered between rounds until oxygen saturation had sufficiently recovered. All procedures, including arterial blood sampling, were conducted under the supervision of trained medical personnel, including an anesthesiologist. Oxygen saturation, heart rate, vital signs, and symptoms associated with hypoxemia or arterial catheterization were continuously monitored. If clinically significant symptoms or vital-sign abnormalities occurred, the procedure was discontinued immediately, 100% oxygen was administered, and appropriate treatment was provided. All adverse events were documented and classified as mild, moderate, or severe according to their functional impact and the required medical intervention.

**Figure 2:**
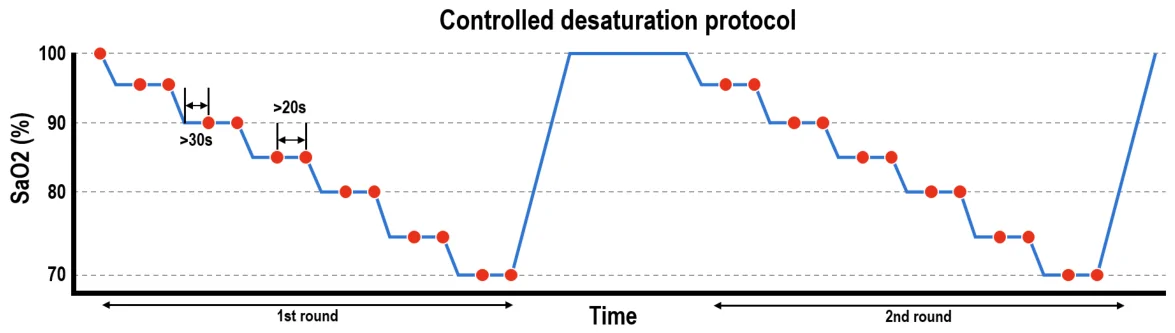
Controlled desaturation protocol and arterial blood sampling schedule. Six target oxygen saturation plateaus (94, 90, 85, 80, 75, and 70%) were sequentially induced during each of two desaturation rounds, with recovery to normoxia between rounds. At each plateau, two arterial blood samples were collected: the first at least 30 s after stabilization and the second after an additional interval of at least 20 s. The blue line indicates the target oxygen saturation, and the red circles indicate arterial blood sampling time points.

A total of 25 arterial blood samples, corresponding to approximately 50 mL of blood, were collected from each participant. These comprised one sample obtained during baseline normoxia (S1), 12 samples collected during the first desaturation round (S2–S13), and 12 samples collected during the second round (S14–S25). During each round, two arterial blood samples were obtained at each of the six target plateaus, as illustrated in Figure 2. Sampling was initiated only after both the breath-by-breath SaO_2_ estimated using the Severinghaus equation and the SpO_2_ measured using the N-560 had stabilized within the target range. The first arterial blood sample at each plateau was collected at least 30 s after the target plateau had been reached, and consecutive samples obtained at the same plateau were separated by an interval of at least 20 s.

### 2.5. Overall Processing Steps

**Figure 3:**
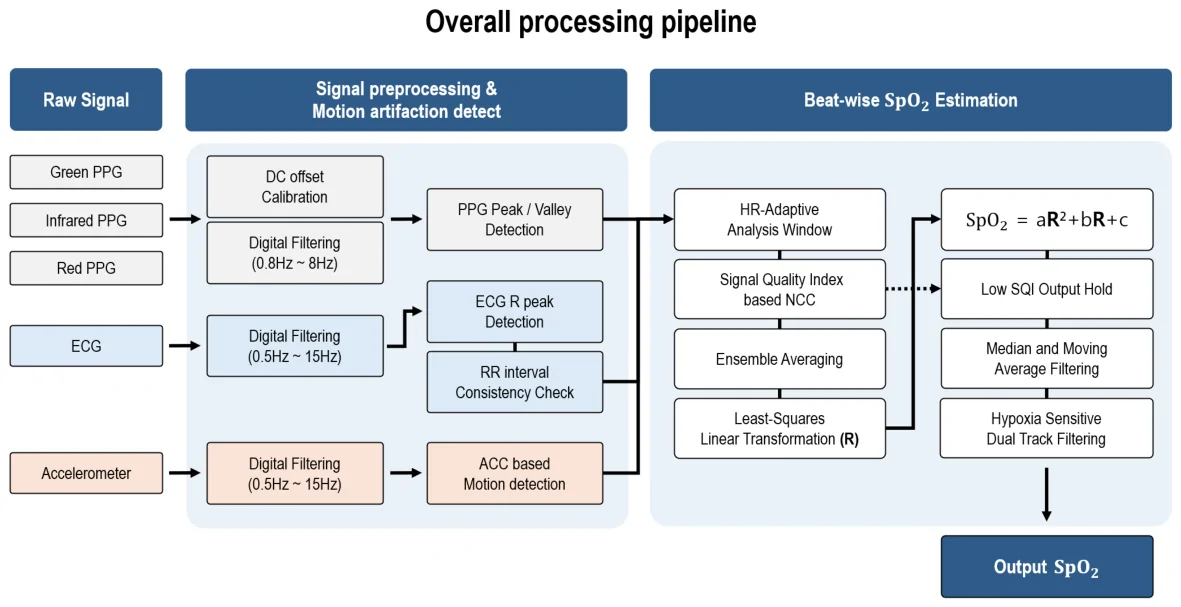
Overall pipeline of the proposed chest reflectance PPG-based SpO_2_ estimation algorithm. Raw green, red, and IR PPG signals, together with accelerometer data, are first processed by DC offset calibration, filtering, and motion detection. Green PPG is used for peak detection, pulse-rate estimation, and RR-interval consistency checking. Beat-wise SpO_2_ estimation is then performed using an HR-adaptive analysis window, NCC-based signal quality assessment, beat ensemble averaging, least-squares AC estimation, and calibration of the ratio-of-ratios value. The final SpO_2_ output is generated after low-SQI output hold, median and moving-average filtering, and hypoxia-sensitive dual-track post-processing.

The overall SpO_2_ estimation pipeline is illustrated in Figure 3. All processing stages were designed to minimize computational complexity and output latency, enabling real-time SpO_2_ monitoring. The M350 simultaneously acquired green, red, and IR PPG signals, together with ECG, accelerometer, and respiration signals. However, the respiration signal was not used in the proposed algorithm. The acquired signals underwent preprocessing, PPG and ECG peak detection, motion artifact detection, HR-adaptive windowing, and normalized cross-correlation-based signal quality assessment. For valid PPG beats, ensemble averaging and least-squares-based AC component estimation were applied to calculate the *R*. Beat-wise SpO_2_ values were then obtained using the calibration equation followed by post-processing.

#### 2.5.1. Signal Preprocessing

The green, red, and IR PPG signals and the ECG signal were acquired at a sampling rate of 125 Hz. All subsequent signal processing was performed at the original sampling rate without resampling. DC offset calibration was first applied to the raw PPG signals to correct channel-specific baselines. In chest reflectance PPG, the DC level of each wavelength channel may vary depending on sensor–skin contact, skin-surface curvature, sensor attachment pressure, ambient light, and differences in the optical paths between the LEDs and photodiode [17,18]. Because these channel-dependent DC offsets may affect AC component estimation and comparisons among wavelength channels, the baseline of each channel was corrected before subsequent signal processing.

Following DC offset calibration, sequential digital filtering was performed to obtain waveforms suitable for beat-wise signal analysis and SpO_2_ estimation. Previous studies have reported that band-pass filtering within the 0.5–8 Hz range effectively suppresses baseline wander and high-frequency components that are not relevant to PPG analysis, whereas moving-average filtering is useful for reducing short-term signal fluctuations [17,19]. In pulse oximetry, the filter bandwidth of the photodiode signal may affect not only noise suppression but also pulse-waveform morphology and ratio-of-ratios-based SpO_2_ estimation. Therefore, preprocessing is required to remove unwanted frequency components while preserving the pulsatile component [20].

In this study, a second-order finite impulse response (FIR) high-pass filter with a cutoff frequency of 0.8 Hz was applied to the PPG signals to suppress low-frequency drift caused by respiratory variation, changes in sensor–skin contact, and baseline wander. A second-order FIR band-stop filter centered at 60 Hz was subsequently applied to remove power-line interference [21]. A second-order FIR low-pass filter with a cutoff frequency of 8 Hz was then applied to attenuate noise at frequencies above the pulsatile component [19,21]. Finally, a 25-point moving-average filter was applied to reduce short-term signal fluctuations and provide stable input signals for subsequent beat-wise analysis and SpO_2_ estimation [19]. The ECG signal was processed using a 0.5–15 Hz band-pass filter to suppress baseline drift and high-frequency noise while preserving the cardiac waveform required for subsequent R-peak detection.

#### 2.5.2. Motion Artifact Detection

In chest-worn reflectance PPG, body movement, respiratory chest-wall motion, changes in sensor attachment pressure, and variations in sensor–skin contact can distort the PPG waveform [3,22]. These disturbances can destabilize the pulsatile components of the red and IR PPG signals, thereby affecting the accuracy of peak and valley detection and AC component estimation. Moreover, failure to adequately detect movement artifacts in pulse oximetry recordings may cause transient decreases in SpO_2_ to be misinterpreted as hypoxemia, even in the absence of true oxygen desaturation [23]. Therefore, an accelerometer (ACC)-based motion artifact detection step was incorporated into the proposed algorithm to improve the reliability of SpO_2_ estimation.

Triaxial ACC signals ($a_{x}$, $a_{y}$, and $a_{z}$) were acquired at a sampling rate of 25 Hz and processed at the original sampling rate without resampling. A second-order IIR band-pass filter, implemented in direct-form II transposed, was applied to the raw signal from each axis, with lower and upper cutoff frequencies of 0.5 Hz and 3 Hz, respectively. This filtering step was performed to suppress the gravitational component and low-frequency variations associated with gradual postural changes while retaining dynamic acceleration components caused by body movement, upper-body motion, and changes in sensor contact. The vector magnitude of the filtered triaxial ACC signals was then calculated and used as a single motion reference signal with reduced dependence on sensor orientation [24,25]:

(1) $$A_{\mathrm{mag}}\left(n\right)=\sqrt{a_{x}{\left(n\right)}^{2}+a_{y}{\left(n\right)}^{2}+a_{z}{\left(n\right)}^{2}}.$$

Here, $a_{x}\left(n\right)$, $a_{y}\left(n\right)$, and $a_{z}\left(n\right)$ represent the filtered acceleration signals along the three axes at the *n*th sample. The difference in acceleration magnitude between consecutive samples was calculated and used as a signed motion index reflecting rapid changes in movement [26]. When the motion index fell outside the range −20.06 to +20.06 ADC counts for five consecutive samples, the motion flag was set and the corresponding interval was classified as containing motion artifacts; the flag was cleared after five consecutive samples within the range.

Intervals classified as containing motion artifacts were excluded from subsequent beat validation and SpO_2_ estimation. During real-time monitoring, updates of the SpO_2_ estimate were suspended while motion artifacts were detected, and the most recent valid SpO_2_ value was temporarily retained. This procedure was intended to reduce transient SpO_2_ output errors caused by body movement and changes in sensor contact.

#### 2.5.3. PPG Peak Detection

PPG-based SpO_2_ estimation requires reliable segmentation of the red and IR PPG signals into individual cardiac cycles. In this study, the green PPG signal was used as the reference signal for defining the beat-wise analysis windows. Because green light has a shorter wavelength than red and IR light, green PPG is relatively more sensitive to pulsatile blood-volume changes in superficial vascular layers and is therefore advantageous for cardiac-cycle detection [27,28,29]. Green PPG has also been used as a reference waveform for beat-quality assessment and outlier rejection of red and IR PPG signals in a previous study of sternal reflectance pulse oximetry [15]. A recent study of 24 healthy participants also reported higher green-wavelength PPG signal quality than that of red and IR PPG at core-body sites, including the sternum, supporting the use of green PPG as a reference waveform for chest-based beat detection [30]. In contrast, although red and IR PPG signals are essential for SpO_2_ estimation because they reflect differences in light absorption between oxygenated and deoxygenated hemoglobin, their pulsatile components may have low amplitudes at low-perfusion reflectance measurement sites such as the chest. Consequently, peak and valley detection may become unstable because of respiratory and motion-related disturbances [15]. Therefore, the beat locations detected from the green PPG signal were used to align the red and IR PPG signals into corresponding cardiac cycles.

To quantitatively compare pulsatile signal amplitude across wavelengths, the perfusion index (PI) was calculated for green, red, and IR PPG signals in all 12 participants during the controlled desaturation protocol. Signals were analyzed in 10-s windows with 5-s overlap. For each channel, the DC component was defined as the mean of the DC-corrected signal, whereas the AC component was estimated from the 0.5–5 Hz band-pass-filtered signal as the 95th–5th percentile difference. PI was calculated as 100×AC/DC. Windows containing signal saturation, low green-channel pulse-spectrum SNR, or PI values exceeding 20% were excluded. For each participant, the median PI across valid windows was calculated for each wavelength, and green-versus-red and green-versus-IR differences were evaluated using paired Wilcoxon signed-rank tests. This supplementary analysis was performed to provide quantitative support for the predefined use of green PPG as the reference channel for cardiac-cycle detection.

**Figure 4:**
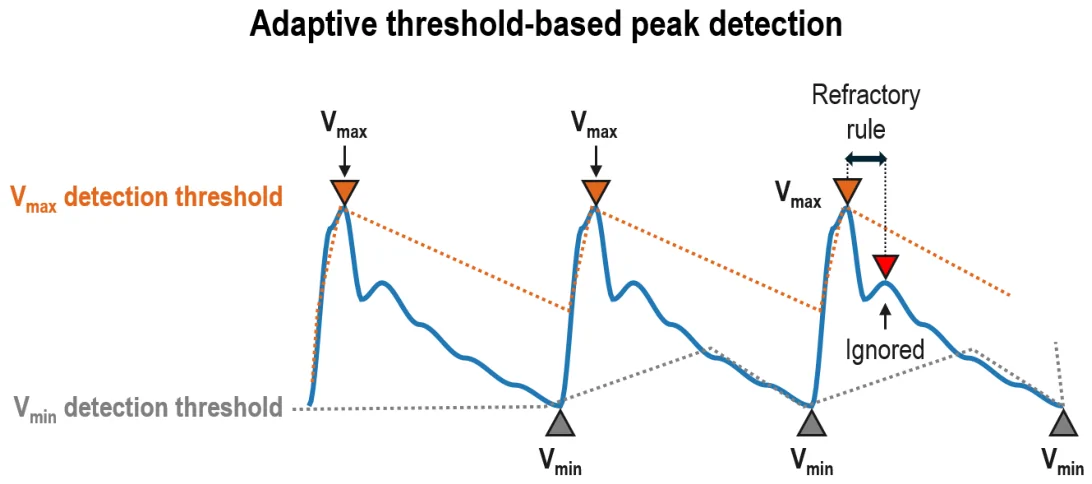
Adaptive threshold-based PPG peak and valley detection. The dynamic thresholds for $V_{\max}$ and $V_{\min}$ track the PPG waveform to identify candidate peaks and valleys. Candidate detections occurring within the refractory period are rejected to prevent physiologically implausible duplicate beat detections.

For beat detection, peaks and valleys in the green PPG signal were detected using an algorithm based on adaptive threshold detection (ADT) [31]. ADT identifies candidate peaks and valleys using a threshold that is dynamically updated according to changes in PPG amplitude. As illustrated in Figure 4, the threshold was designed to track the waveform, and points at which the direction of the signal changed were identified as candidate peaks or valleys. This approach is more robust to baseline and amplitude variations than fixed-threshold or simple local-extrema detection.

To enable real-time processing, the detection algorithm was implemented in a sample-by-sample manner using only the current input sample and state variables updated from the preceding beat. An adaptive refractory rule was applied to remove duplicate detections occurring at short intervals: rather than a fixed post-detection blanking interval, a candidate beat was rejected when the resulting R-R interval fell outside 0.50 to 1.50 times the running mean R-R interval, so that the effective refractory limit adapts to the prevailing pulse rate; before a running mean was established, absolute limits of 0.5 and 1.5 s (120 and 40 bpm) were applied. Each candidate location was corrected by searching for the actual local maximum or minimum within a limited surrounding interval. If no beat was detected for a predefined period, a back-search was performed within a limited interval based on the most recent valid pulse interval. Intervals classified as unreliable by motion artifact detection or the signal quality index (SQI) were excluded from the final SpO_2_ calculation. The resulting green PPG beat locations were used to define the beat-wise analysis windows for the red and IR PPG signals.

#### 2.5.4. ECG R-Peak Detection

The ECG signal was used as an auxiliary signal to assess the temporal consistency of green PPG-based beat detection and to provide reference heart-rate information for defining the HR-adaptive analysis window. Motion-induced changes in blood volume at the measurement site can cause motion artifacts and pulse-detection errors in reflectance PPG [32], and ECG R-peaks have been used as reference fiducial points for evaluating PPG peak-detection performance [33]. Cardiac rhythm information derived from ECG has also been applied to the processing and reconstruction of PPG signals affected by motion artifacts [34]. Based on these findings, heart-rate information derived from ECG R-peaks was used as an auxiliary reference to assess the plausibility of PPG beat timing and to establish stable analysis windows.

For integration into the real-time beat-wise SpO_2_ estimation framework, an ECG R-peak detection algorithm was implemented based on an angle-based QRS detection method [35]. This method identifies candidate R-peak regions using the rapid slope changes characteristic of the QRS complex. For each incoming ECG sample, slope-based features and an adaptive threshold were sequentially updated, and regions exceeding the threshold were identified as R-peak candidates. The final R-peak location was then refined by searching for the maximum amplitude of the original ECG signal within a limited local interval surrounding each candidate. All operations were performed in a sample-by-sample manner using a fixed-length buffer to minimize computational burden and processing latency.

Instantaneous heart rate was calculated from the detected ECG R-peaks and used to assess the physiological plausibility and temporal consistency of the pulse intervals detected from the green PPG signal. ECG R-peaks were not directly used to estimate the AC or DC components of the red and IR PPG signals; rather, they served as an auxiliary reference for defining the HR-adaptive analysis window and identifying abnormal PPG beat intervals. This approach reduced the influence of low perfusion, respiratory modulation, and transient waveform distortion on PPG beat segmentation, thereby providing stable analysis intervals for real-time beat-wise SpO_2_ estimation.

#### 2.5.5. HR-Adaptive Analysis Window

Reliable definition of the analysis interval corresponding to each pulse cycle is essential for PPG-based SpO_2_ estimation. In conventional pulse oximetry, the pulsatile AC components are typically estimated by directly detecting peaks and valleys in the red and IR PPG signals [4,5]. However, at low-perfusion reflectance measurement sites such as the chest, the pulsatile amplitudes of the red and IR PPG signals are relatively small, and the waveforms are readily distorted by respiratory motion and changes in sensor–skin contact. Consequently, reliable peak and valley detection is difficult [15,16,17]. Therefore, rather than directly detecting peaks in the red and IR PPG signals, the proposed method defined an analysis window for each pulse cycle using peaks detected from the green PPG signal, which exhibited a more distinct pulsatile component.

The interval between consecutive ECG R-peaks was defined as the R-R interval, whereas the interval between consecutive green PPG peaks was defined as the pulse interval. Whenever a new green PPG peak was detected, the pulse interval and pulse rate were calculated from the time difference relative to the preceding peak. A buffer containing recent pulse intervals was continuously updated and used to assess the temporal plausibility of each newly detected peak. If a pulse interval fell outside the predefined physiological range or was inconsistent with the recent pulse-interval pattern, the corresponding peak was classified as an artifact or false detection and excluded from subsequent SpO_2_ estimation.

Each valid green PPG peak was used as a reference point for constructing the HR-adaptive analysis window. Rather than using a fixed window length, the window was dynamically adjusted according to the current pulse rate and recent pulse intervals. A shorter window was applied when the pulse interval decreased, whereas a longer window was applied when the pulse interval increased, thereby ensuring that the corresponding red and IR PPG segments were reliably included within each pulse cycle. The window duration was set to 1.4 times the current pulse interval and constrained to 80–220 samples (0.64–1.76 s at 125 Hz) to prevent physiologically implausible window lengths.

Whereas a previous study applied a fixed analysis-window length based on the mean R-R interval over the entire recording session [15], the proposed method updated the window length according to real-time changes in the pulse interval. This approach accounted for changes in heart rate and transient variations in pulse interval while allowing the AC and DC components of the red and IR PPG signals to be estimated over corresponding pulse cycles. Within each analysis window, the DC component of each wavelength was calculated as the mean of the low-pass-filtered signal. AC component estimation and *R* calculation were performed only for windows that satisfied the signal-quality criteria.

#### 2.5.6. Signal Quality Index Based on Normalized Cross-Correlation

A normalized cross-correlation (NCC)-based SQI was calculated to determine whether each PPG beat segmented using the HR-adaptive analysis window was suitable for SpO_2_ estimation [15,36,37]. In chest reflectance PPG, the red and IR waveforms may be transiently distorted by low perfusion, body movement, respiratory motion, or changes in sensor–skin contact. Therefore, the NCC-based SQI was used as a beat-wise quality-control metric.

The green PPG signal, which was relatively stable under low-perfusion conditions, was used as the reference waveform. Waveform similarity between the green PPG and the red or IR PPG signal was evaluated within the same beat window [38]. Because NCC quantifies morphological similarity while reducing the influence of differences in absolute amplitude among channels, it was used to determine whether beat morphology was preserved in each wavelength channel. The mean-centered waveforms within each beat window were defined as

(2) $$x_{\lambda }\left(n\right)={\mathrm{PPG}}_{\lambda }\left(n\right)-\bar{{\mathrm{PPG}}_{\lambda }},$$

(3) $$g_{k}\left(n\right)={\mathrm{PPG}}_{\mathrm{green}}\left(n+k\right)-\bar{{\mathrm{PPG}}_{\mathrm{green}}}.$$

Here, λ∈{red,IR} denotes the PPG channel, *n* denotes the sample index within the beat window, *N* denotes the number of samples in the window, and *k* denotes the lag used to compensate for small temporal offsets between the green PPG and the red or IR PPG signal. $\bar{{\mathrm{PPG}}_{\lambda }}$ and $\bar{{\mathrm{PPG}}_{\mathrm{green}}}$ represent the corresponding mean values within the beat window. The NCC between the PPG beat at wavelength λ and the green PPG reference beat was then calculated. To account for small temporal offsets among the red, IR, and green PPG signals, NCC was evaluated over lags ranging from −8 to +8 samples, corresponding to −64 to +64 ms. The maximum NCC value within this range was used as the SQI for the corresponding beat:

(4) $${\mathrm{NCC}}_{k,\lambda }=\frac{\sum_{n=1}^{N}x_{\lambda }\left(n\right)g_{k}\left(n\right)}{\sqrt{\left(\sum_{n=1}^{N}x_{\lambda }^{2}\left(n\right)\right)\left(\sum_{n=1}^{N}g_{k}^{2}\left(n\right)\right)}},$$

(5) $${\mathrm{SQI}}_{\lambda }=\max_{k\in [-8,8]}\left({\mathrm{NCC}}_{k,\lambda }\right).$$

Only beats for which both the red and IR PPG SQI values were equal to or greater than the predefined threshold were classified as valid and included in ensemble averaging, AC component estimation, and *R* calculation. If the SQI of either channel was below the threshold, the corresponding beat was considered potentially distorted by body movement, respiratory motion, or unstable sensor contact and was excluded from subsequent processing.

Beats that did not satisfy the SQI criterion were not included in the accumulation of valid windows for ensemble averaging. During these periods, updates of the SpO_2_ estimate were suspended, and the most recent valid output was temporarily retained. If poor signal quality persisted beyond a predefined allowable duration, the accumulated valid windows were reset and the SpO_2_ output was marked as invalid. Once both the red and IR PPG signals again satisfied the SQI criterion, valid windows were accumulated anew and SpO_2_ estimation was resumed. This beat-wise quality-control procedure prevented distorted PPG segments from being included in the ratio-of-ratios calculation and improved the stability of real-time SpO_2_ output.

#### 2.5.7. SpO_2_ Estimation Using Least-Squares Linear Transformation

SpO_2_ estimation was performed using only red and IR PPG beats that were segmented within HR-adaptive analysis windows and satisfied the NCC-based SQI criterion. Four consecutive valid beat windows were accumulated and ensemble-averaged to obtain representative red and IR PPG waveforms. This procedure was applied to suppress random noise and transient beat-to-beat waveform variability before estimating the AC amplitude ratio. The ensemble size was determined through a preliminary comparison of different numbers of beats, and a four-beat ensemble was selected as a compromise between noise reduction and temporal responsiveness.

**Figure 5:**
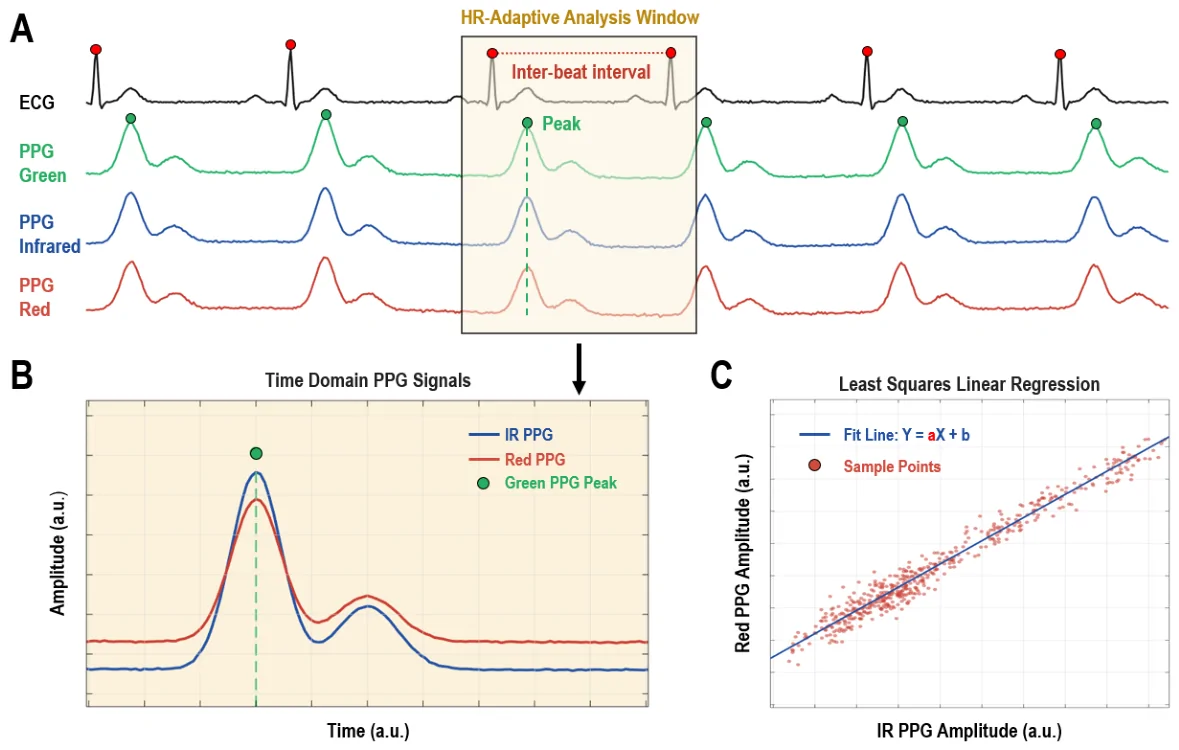
Key steps in chest reflectance PPG-based SpO_2_ estimation. (**A**) Simultaneously recorded ECG and green, red, and IR PPG signals. Green PPG peaks define the HR-adaptive analysis windows, while ECG R-peaks provide reference cardiac timing. The green dashed line marks the green PPG peak. (**B**) Beat-aligned IR and red PPG waveforms extracted within each analysis window and combined by ensemble averaging. (**C**) Least-squares linear regression between the averaged IR and red PPG samples to estimate their relative pulsatile amplitude ratio for SpO_2_ calculation.

To reduce the dependence of AC estimation on individual peak and valley locations, a least-squares-based linear transformation was applied [15,39]. In chest-worn reflectance PPG, local peaks and valleys may be distorted by low perfusion, respiratory motion, and variations in sensor–skin contact, thereby increasing the variability of peak-to-valley-based AC estimates. Accordingly, the relative amplitude relationship between the red and IR PPG waveforms was estimated using all samples within each ensemble-averaged window. As illustrated in Figure 5C, a linear regression of the red PPG amplitude on the corresponding IR PPG amplitude was performed, and the slope of the fitted line was used as the red-to-IR AC amplitude ratio.

The ensemble-averaged red and IR PPG waveforms were denoted by ${\mathrm{PPG}}_{\mathrm{red}}\left(n\right)$ and ${\mathrm{PPG}}_{\mathrm{IR}}\left(n\right)$, respectively. The linear relationship between the two waveforms, the least-squares parameter estimates, the wavelength-specific DC components, and the final ratio-of-ratios, *R*, were defined as follows:

(6) $$\begin{array}{cc} \;{\mathrm{PPG}}_{\mathrm{red}}\left(n\right)\; & \;\approx \alpha_{1}{\mathrm{PPG}}_{\mathrm{IR}}\left(n\right)+\alpha_{2}, \end{array}$$

(7) $$\begin{array}{cc} \;(\alpha_{1}^{*},\alpha_{2}^{*})\; & =\underset{\alpha_{1},\alpha_{2}}{\arg\min}\sum_{n=1}^{N}{\left[{\mathrm{PPG}}_{\mathrm{red}}\left(n\right)-\alpha_{1}{\mathrm{PPG}}_{\mathrm{IR}}\left(n\right)-\alpha_{2}\right]}^{2}, \end{array}$$

(8) $$\begin{array}{cc} \;\frac{AC_{\mathrm{red}}}{AC_{\mathrm{IR}}}\; & =\alpha_{1}^{*}, \end{array}$$

(9) $$\begin{array}{cc} \;DC_{\mathrm{red}}\; & =\frac{1}{N}\sum_{n=1}^{N}{\mathrm{PPG}}_{\mathrm{red}}\left(n\right),\;DC_{\mathrm{IR}}=\frac{1}{N}\sum_{n=1}^{N}{\mathrm{PPG}}_{\mathrm{IR}}\left(n\right),\; \end{array}$$

(10) $$\begin{array}{cc} \;R\; & =\frac{AC_{\mathrm{red}}/DC_{\mathrm{red}}}{AC_{\mathrm{IR}}/DC_{\mathrm{IR}}}=\frac{\alpha_{1}^{*}}{DC_{\mathrm{red}}/DC_{\mathrm{IR}}}. \end{array}$$

Here, $\alpha_{1}^{*}$ represents the scale factor between the pulsatile components of the red and IR PPG waveforms and was therefore used as the red-to-IR AC amplitude ratio. The parameter $\alpha_{2}^{*}$ is the intercept term that accounts for differences in baseline level between the two waveforms, and *N* denotes the number of samples within the analysis window. The DC component at each wavelength was calculated as the mean value of the corresponding ensemble-averaged PPG waveform.

The resulting *R* value was subsequently converted to SpO_2_ using the calibration equation. By estimating the relative AC amplitude from the entire waveform rather than from individual peaks and valleys, the proposed approach was designed to reduce sensitivity to local peak distortion, valley drift, and residual noise. Restricting the analysis to SQI-qualified beats and applying four-beat ensemble averaging further limited the influence of low-quality beats and transient waveform variations on the estimation of *R*.

#### 2.5.8. SpO_2_ Calibration Equation Estimation

A polynomial calibration equation was applied to convert the calculated *R* into SpO_2_. The relationship between *R* and SpO_2_ in pulse oximetry is not strictly linear, and the nonlinearity may become more pronounced at lower oxygen saturation levels. Accordingly, first-, second-, and third-order polynomial calibration models were fitted and compared. The goodness of fit of each model was evaluated using the fit root-mean-square error (fit RMSE), defined as the root-mean-square difference between the reference SaO_2_ measured using an arterial blood gas analyzer and the SpO_2_ predicted by applying the corresponding calibration equation to the *R* values obtained from the M350. Because fit RMSE generally decreases as the polynomial order increases, it was not used as the sole criterion for model selection.

To assess model generalizability, subject-level leave-one-subject-out (LOSO) cross-validation was additionally performed. In each iteration, the calibration equation was fitted using data from four of the five participants and evaluated using data from the remaining participant. This procedure was repeated until each participant had served once as the validation subject, and the mean and standard deviation of the subject-specific RMSE values were calculated for each polynomial order. The final calibration equation was selected by jointly considering the fit RMSE and LOSO RMSE, whether the model preserved a physiologically plausible monotonically decreasing relationship between *R* and SpO_2_ over the observed *R* range, the stability of the estimated coefficients under the small-sample condition, and model parsimony.

#### 2.5.9. SpO_2_ Post-Processing

Post-processing was applied to the final SpO_2_ estimates to suppress residual noise and abnormal spikes. When the lower of the red and IR PPG SQI values was below 0.65, the corresponding SpO_2_ estimate was discarded, and the most recent valid output was retained using an SQI-freeze rule. No maximum holding duration was imposed on the retained SpO_2_ value in the current implementation; the most recent valid value therefore remained displayed until a subsequent SpO_2_ estimate satisfied the SQI criterion. The SQI threshold of 0.65 was empirically selected during algorithm development. Although previous studies used an NCC-based threshold of 0.70 [15,40], preliminary evaluation showed that this value resulted in excessive rejection of otherwise usable beats; therefore, 0.65 was fixed before validation and was not optimized using the validation dataset. A median filter and a moving-average filter were then applied sequentially, and the degree of smoothing was adjusted by adapting their window lengths according to the prevailing saturation level. This dual-track filtering strategy was designed to balance output stability against responsiveness to hypoxemic changes. Under normal conditions, both window lengths were set to 5 beats. When SpO_2_ remained at or below 88% for at least three consecutive beats, the condition was classified as hypoxemia and both window lengths were reduced to 3 beats, allowing more rapid tracking of changes in oxygen saturation; the default lengths were restored once SpO_2_ remained at or above 93% for five consecutive beats.

Because post-processing was performed on beat-wise SpO_2_ estimates, the temporal span of the filtering windows varied with heart rate rather than being directly determined by the 125-Hz PPG sampling rate. Consequently, the effective response time of the complete post-processing pipeline was expected to vary with heart rate and was not independently quantified in seconds in the present study. This adaptive post-processing strategy was designed to maintain output stability in chest-worn measurements affected by low perfusion and motion while preserving responsiveness to clinically important changes in SpO_2_.

### 2.6. Evaluation

The performance of the proposed SpO_2_ estimation algorithm was evaluated using paired samples obtained from the second-stage controlled desaturation study. The sample size was determined in accordance with ISO 80601-2-61:2017, which requires a minimum of 10 healthy participants and at least 200 valid data pairs distributed across the target saturation range. For each sample, the SpO_2_ estimated from the M350 was compared with the reference SaO_2_ measured using an arterial blood gas analyzer. The accuracy root mean square ($A_{\mathrm{rms}}$), which is commonly used to assess pulse oximeter accuracy, was calculated as the primary performance metric. The residual standard error ($S_{\mathrm{res}}$) was also calculated to quantify the dispersion of the estimates around the fitted relationship. The two metrics were defined as follows:

(11) $$\begin{array}{cc} A_{\mathrm{rms}}\; & =\sqrt{\frac{1}{n}\sum_{i=1}^{n}{\left({\mathrm{SpO}}_{2,M350,i}-{\mathrm{SaO}}_{2,\mathrm{ref},i}\right)}^{2}}, \end{array}$$

(12) $$\begin{array}{cc} \;{\mathrm{SpO}}_{2,\mathrm{fit},i}\; & ={\hat{\beta }}_{0}+{\hat{\beta }}_{1}{\mathrm{SaO}}_{2,\mathrm{ref},i}, \end{array}$$

(13) $$\begin{array}{cc} \;S_{\mathrm{res}}\; & =\sqrt{\frac{\sum_{i=1}^{n}{\left({\mathrm{SpO}}_{2,M350,i}-{\mathrm{SpO}}_{2,\mathrm{fit},i}\right)}^{2}}{n-2}}. \end{array}$$

Here, ${\mathrm{SpO}}_{2,M350,i}$ denotes the SpO_2_ value estimated by the proposed algorithm, and ${\mathrm{SaO}}_{2,\mathrm{ref},i}$ denotes the corresponding reference arterial oxygen saturation measured using an arterial blood gas analyzer. A linear regression model was fitted using reference SaO_2_ as the independent variable and M350-derived SpO_2_ as the dependent variable. The fitted value was calculated as ${\mathrm{SpO}}_{2,\mathrm{fit},i}={\hat{\beta }}_{0}+{\hat{\beta }}_{1}{\mathrm{SaO}}_{2,\mathrm{ref},i}$. The residual standard error, $S_{\mathrm{res}}$, represents the dispersion of the M350-derived SpO_2_ values around the fitted regression line.

Because each participant contributed multiple paired measurements rather than a single independent observation, the point estimates of $A_{\mathrm{rms}}$ and mean bias were calculated from the pooled set of valid data pairs. To account for within-participant dependence when estimating statistical uncertainty, 95% confidence intervals (CIs) for $A_{\mathrm{rms}}$ and mean bias were obtained using participant-level cluster bootstrap resampling with 10,000 replicates. In each bootstrap replicate, participants were sampled with replacement, and all repeated measurements from each selected participant were retained together. The 2.5th and 97.5th percentiles of the bootstrap distributions were used as the lower and upper limits of the 95% CIs. In addition, participant was treated as a random factor for variance-component analysis. The variance of the paired SpO_2_–SaO_2_ differences was partitioned into between-participant ($\sigma_{u}^{2}$) and within-participant ($\sigma^{2}$) components using a random-intercept mixed-effects model fitted by restricted maximum likelihood (REML). The intraclass correlation coefficient (ICC) was calculated as $\sigma_{u}^{2}/\left(\sigma_{u}^{2}+\sigma^{2}\right)$. These analyses were performed both over the full reference saturation range and within each nominal saturation range.

## 3. Results

### 3.1. Comparison of PPG Perfusion Index Across Wavelengths

To quantitatively compare wavelength-specific pulsatile amplitude, participant-level median PI values were derived from valid 10-s windows during the controlled desaturation protocol. Across the 12 participants, the median PI was 0.563% [IQR, 0.420–0.780] for green PPG, compared with 0.166% [IQR, 0.133–0.217] for red PPG and 0.158% [IQR, 0.127–0.214] for IR PPG. Green PPG showed significantly higher PI than both red and IR PPG (paired Wilcoxon signed-rank tests, both p<0.001), and the green-channel PI was higher than both other channels in all 12 participants. These findings provide quantitative support for using green PPG as the reference signal for cardiac-cycle detection at the chest.

### 3.2. Calibration Model Comparison and Selection

**Figure 6:**
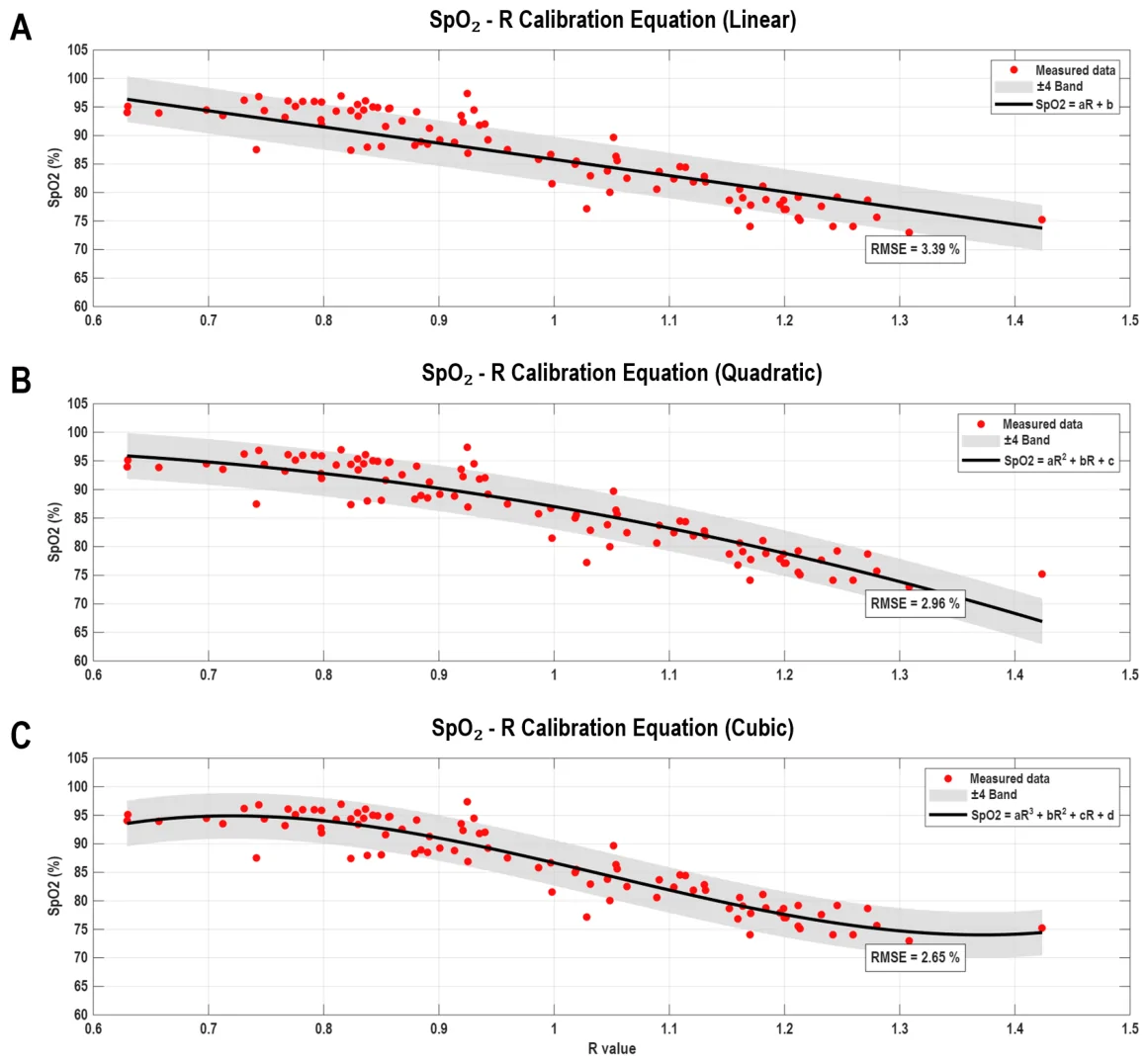
Polynomial calibration models relating *R* to reference ${\mathrm{SaO}}_{2}$. Red circles represent 94 paired measurements from five participants, black lines indicate the fitted calibration curves, and gray shaded areas indicate ±4 percentage points around the fitted curves. The linear (**A**), quadratic (**B**), and cubic models (**C**) yielded fit RMSEs of 3.39%, 2.96%, and 2.65%, respectively. The quadratic model was selected as the final calibration function based on predictive performance, monotonicity, physiological plausibility, and model parsimony. Exact device-specific coefficients are not reported because they constitute proprietary calibration parameters of the investigational device.

No prespecified adverse events occurred during the calibration study, and all participants completed the planned arterial blood sampling procedure. The calibration-development dataset comprised 94 paired $\left(R,{\mathrm{SaO}}_{2}\right)$ observations obtained from the five participants enrolled in Stage 1. No Stage 2 data were used for calibration fitting or model selection. First-, second-, and third-order polynomial models were fitted to characterize the relationship between *R* and reference SaO_2_. The fitted polynomial calibration models are presented in Figure 6. The measured data showed an overall decrease in reference SaO_2_ with increasing *R*, with pronounced nonlinear curvature in the low-saturation region at R>1.1. This trend is consistent with the established nonlinear inverse relationship between *R* and oxygen saturation in pulse oximetry [41,42].

The fit RMSEs for the linear, quadratic, and cubic models were 3.39%, 2.96%, and 2.65%, respectively. In LOSO cross-validation across the five participants, the corresponding mean RMSEs were 3.29±1.48%, 3.19±1.30%, and 2.87±0.91%, respectively. Thus, the cubic model yielded the lowest numerical error for both the fit and LOSO cross-validation.

However, model selection was not based solely on numerical error. Although the cubic model provided the lowest RMSE, it did not preserve a monotonically decreasing relationship over the full calibration range. The slope changed sign at approximately R=0.72 and R=1.37, resulting in an increase in predicted SpO_2_ with increasing *R* when R<0.72 or R>1.37. This behavior is inconsistent with the established inverse optical relationship between *R* and oxygen saturation expected in conventional red/infrared pulse oximetry [41,42,43]. In particular, because the region at R<0.72 corresponds to normal or high oxygen saturation, the non-monotonic behavior in this range reduces the physical plausibility of the cubic calibration function despite its lower numerical error.

The linear model preserved the expected inverse relationship but showed higher fit and LOSO RMSEs than the quadratic model, indicating that the linear model did not adequately capture the nonlinear relationship between *R* and oxygen saturation. In contrast, the quadratic model maintained a monotonically decreasing relationship throughout the observed *R* range while achieving lower fit and LOSO RMSEs than the linear model. Thus, the linear model provided lower predictive performance than the quadratic model, whereas the cubic model achieved lower numerical error at the expense of physically implausible non-monotonic behavior. The quadratic model was the lowest-order model that both improved predictive accuracy relative to the linear model and preserved the expected monotonic inverse relationship throughout the observed calibration range. Therefore, based on the combined consideration of predictive accuracy, physical plausibility, monotonicity, and model parsimony, the quadratic model was selected as the final calibration function. This finalized model was subsequently carried forward unchanged to the Stage 2 pivotal accuracy evaluation, where it was applied to beat-wise SpO_2_ estimation.

### 3.3. Accuracy of the Proposed SpO_2_ Estimation Algorithm

The accuracy of the proposed SpO_2_ estimation algorithm was evaluated using paired samples obtained during the controlled desaturation study. A total of 300 arterial reference samples were collected from 12 participants, with 25 samples obtained from each participant. The first arterial sample (S1) from each participant was collected at baseline before the controlled desaturation procedure and was excluded according to the prespecified analysis criteria. After exclusion of these 12 baseline samples, 288 desaturation samples remained eligible for analysis. An additional 10 samples with reference SaO_2_ values outside the 73–97% range were excluded in accordance with the pooled-data trimming criteria specified in ISO 80601-2-61:2017. These comprised samples S14–S16 from participant 5; S2, S14, and S15 from participant 7; S14 from participant 9; and S2, S3, and S14 from participant 10. Consequently, 278 paired samples were included in the final accuracy analysis.

Physiological circulatory delay was considered when temporally aligning the M350-derived SpO_2_ time series with the reference SaO_2_ values obtained from arterial blood sampling. Changes in oxygen saturation measured by pulse oximetry may lag behind changes in arterial SaO_2_ because of the circulation time to the measurement site and the response characteristics of the measurement system [44,45]. To minimize potential temporal mismatch, each arterial SaO_2_ measurement was paired with the temporally nearest M350-derived SpO_2_ output at the corresponding blood-sampling time. The same temporal-pairing procedure was applied uniformly to all participants, without applying a fixed or participant-specific time shift.

**Figure 7:**
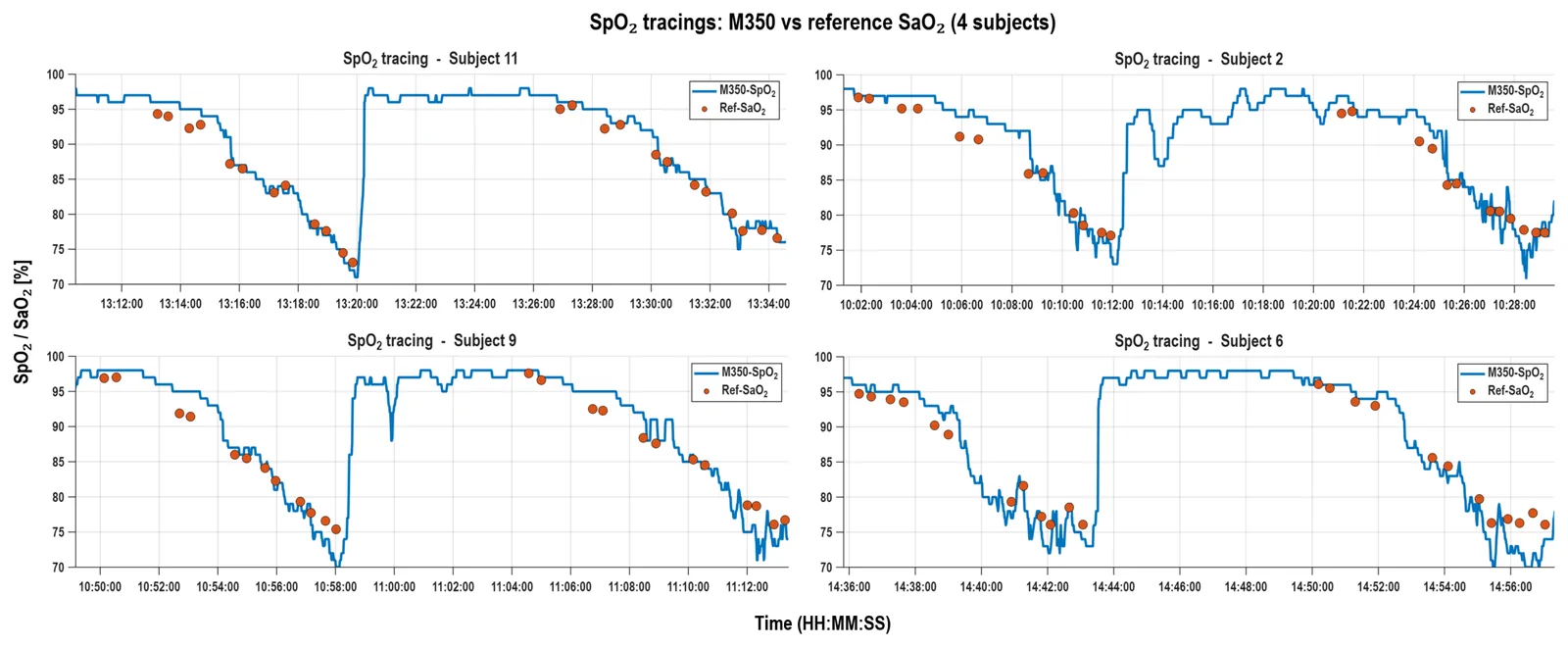
Representative SpO_2_ tracings for four participants (11, 2, 9, and 6). In each panel, the blue line denotes the continuous SpO_2_ measured by the M350 device (M350-SpO_2_) and the orange dots denote the reference arterial SaO_2_ obtained from intermittent blood sampling (Ref-SaO_2_), plotted against clock time (HH:MM:SS).

**Figure 8:**
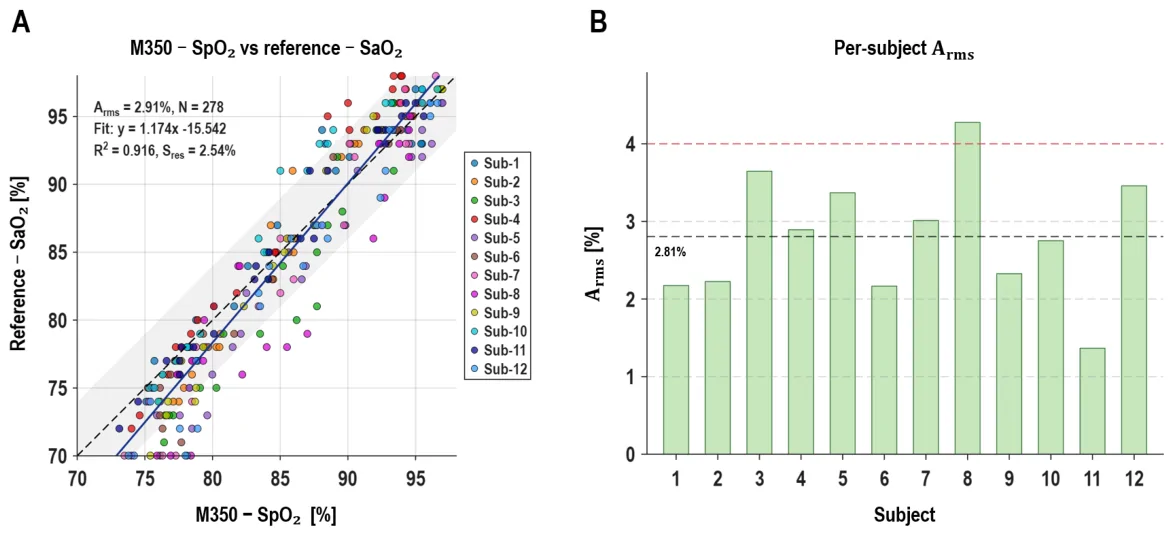
Accuracy of M350-derived SpO_2_ estimates against reference SaO_2_. (**A**) Scatter plot of M350 SpO_2_ versus reference SaO_2_ for all participants. Colors indicate individual participants, the black dashed line represents the identity line (y=x), and the blue solid line represents the linear regression fit, and the gray shaded region represents ±4 percentage points around the identity line. (**B**) Participant-specific $A_{\mathrm{rms}}$ values. The red dashed line indicates the 4% accuracy criterion, and the black dashed line indicates the mean $A_{\mathrm{rms}}$ across participants (2.81%).

Figure 7 shows the temporal changes in M350-derived SpO_2_ during controlled desaturation in four representative participants. The continuously estimated M350 SpO_2_ values followed the stepwise reductions in oxygen saturation, and the reference SaO_2_ values measured at each arterial blood sampling time are superimposed on the same time axis. Figure 8A shows the relationship between M350-derived SpO_2_ and reference SaO_2_ across all 278 paired samples. Each point represents one paired measurement, and the colors indicate individual participants. The black dashed line represents the identity line (y=x), whereas the blue solid line represents the linear regression line fitted to all paired samples. M350-derived SpO_2_ showed a strong linear association with reference SaO_2_ (y=1.174x−15.542, $R^{2}=0.916$), with a $S_{\mathrm{res}}$ of 2.54% around the fitted regression line. Over the trimmed reference SaO_2_ range of 73–97%, the pooled $A_{\mathrm{rms}}$ was 2.91%, satisfying the prespecified accuracy criterion of 4%. Figure 8B presents the participant-specific $A_{\mathrm{rms}}$ values, with a mean across participants of 2.81%.

For the nominal saturation ranges of 70–80%, 80–90%, and 90–100%, the $A_{\mathrm{rms}}$ values were 3.42%, 3.04%, and 2.13%, respectively, with participant-level cluster-bootstrap 95% confidence intervals (CIs) of 2.51–4.17%, 2.35–3.64%, and 1.64–2.66%, respectively (Table 1). The overall $A_{\mathrm{rms}}$ was 2.91% (95% CI, 2.45–3.34%). The overall mean bias was −0.70% (95% CI, −1.58 to 0.15%), whereas the range-specific mean biases were −2.44%, −0.44%, and 0.71%, respectively. These findings indicate that the M350 tended to underestimate reference SaO_2_ at lower saturation levels, whereas a slight overestimation was observed at higher saturation levels.

The 95% CIs for the mean bias and $A_{\mathrm{rms}}$, accounting for repeated measurements within participants, are presented in Table 1. Over the full saturation range, the between-participant variance of the paired SpO_2_–SaO_2_ differences was $2.39\;{\%}^{2}$ (SD, 1.55%), whereas the within-participant variance was $5.81\;{\%}^{2}$ (SD, 2.41%). The corresponding ICC was 0.29, indicating that approximately 71% of the total variability arose within participants.

Table 2 summarizes these participant-specific results in detail. Each participant contributed 21–24 data pairs spanning a comparable portion of the reference range (73.1–97.0% overall). Per-participant $A_{\mathrm{rms}}$ ranged from 1.37% to 4.28% and per-participant bias from −2.86% to +1.87%. On average, 74.6% of each participant’s pairs agreed with the reference within ±3% (range 47.6–95.8%). Weighting each participant equally rather than pooling the data pairs yielded a mean bias of −0.70±1.63% and a mean $A_{\mathrm{rms}}$ of 2.81±0.80%; because $A_{\mathrm{rms}}$ is a root-mean-square quantity, its participant-weighted analogue is the quadratic mean of the per-participant values, which is 2.91% and therefore identical to the pooled estimate.

### 3.4. Bland–Altman Analysis

**Figure 9:**
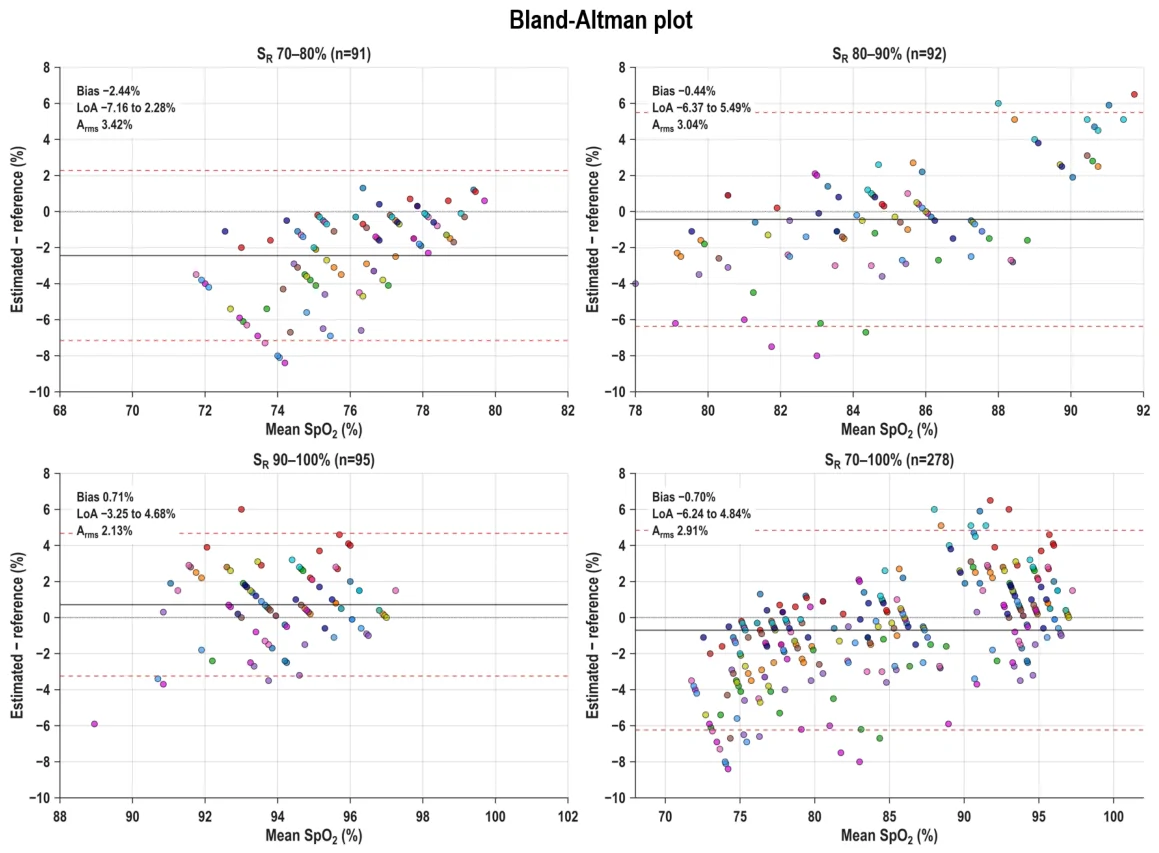
Bland–Altman analysis of agreement between M350-derived SpO_2_ and reference SaO_2_. Results are presented for the 70–80%, 80–90%, 90–100%, and overall 70–100% reference saturation ranges. Each point represents one paired measurement, with colors indicating individual participants. The x-axis represents the mean of the M350 SpO_2_ and reference SaO_2_, and the y-axis represents their difference (M350 SpO_2_ minus reference SaO_2_). Bias denotes the mean difference between the two measurements, LoA denotes the 95% limits of agreement calculated as the mean bias ±1.96 standard deviations, and RMS denotes the root-mean-square error. The solid black line indicates the mean bias, the red dashed lines indicate the 95% limits of agreement, and the black dotted line indicates zero difference.

Agreement between M350-derived SpO_2_ and reference SaO_2_ was assessed using Bland–Altman analysis of the 278 paired samples included in the accuracy analysis. For each paired sample, the mean of the two measurements was plotted on the x-axis, and their difference ($M350\;{\mathrm{SpO}}_{2}-\mathrm{reference}\;{\mathrm{SaO}}_{2}$) was plotted on the y-axis (Figure 9). Across the overall analysis range, the mean bias was −0.70%, and the standard deviation of the differences was 2.83%, yielding 95% limits of agreement from −6.24% to 4.84%. Thus, M350-derived SpO_2_ was, on average, 0.70 percentage points lower than reference SaO_2_. For the nominal saturation ranges of 70–80%, 80–90%, and 90–100%, the mean biases were −2.44%, −0.44%, and 0.71%, respectively, with corresponding 95% limits of agreement of −7.16% to 2.28%, −6.37% to 5.49%, and −3.25% to 4.68%, respectively. These findings indicate that underestimation was more pronounced at lower saturation levels, whereas a slight overestimation was observed at higher saturation levels.

## 4. Discussion

In this study, PPG-based SpO_2_ estimation, which has conventionally been performed at peripheral sites such as the finger, earlobe, and wrist, was extended to chest-worn reflectance PPG and implemented as a real-time algorithm for a wearable device. The chest is well suited for the simultaneous acquisition of ECG, respiration, and motion signals using a single patch-type device. However, compared with peripheral sites, chest PPG is characterized by lower perfusion and greater susceptibility to respiratory and motion artifacts, which limits conventional approaches that estimate the AC components by directly detecting peaks and valleys in the red and IR PPG waveforms. To address these challenges, the proposed SpO_2_ estimation pipeline integrated green PPG-based beat detection, auxiliary validation using ECG R-peaks, HR-adaptive analysis windows, an NCC-based SQI, least-squares estimation of the AC amplitude ratio, and real-time post-processing.

The proposed algorithm was evaluated against reference SaO_2_ measured using an arterial blood gas analyzer during a controlled desaturation study. Over the trimmed reference SaO_2_ range of 73–97%, the overall $A_{\mathrm{rms}}$ was 2.91%, satisfying the 4.0% accuracy criterion specified in ISO 80601-2-61:2017. The $A_{\mathrm{rms}}$ values for the nominal saturation ranges of 70–80%, 80–90%, and 90–100% were 3.42%, 3.04%, and 2.13%, respectively. Although the estimation error increased at lower saturation levels, it remained within 4.0% in all three ranges. Bland–Altman analysis showed a small overall mean bias of −0.70%, although underestimation was more pronounced at lower saturation levels. Importantly, the 95% limits of agreement ranged from −6.24% to +4.84%, indicating that individual SpO_2_ estimates could deviate from arterial SaO_2_ by more than the overall $A_{\mathrm{rms}}$ alone suggests. This combination of a small mean bias and relatively wide limits of agreement indicates limited systematic error but appreciable variability among individual measurements. The median absolute error was 1.65%, and 74.8% of all paired samples fell within ±3% of the reference. The dispersion was also not uniform across the measurement range, with the observed proportional bias indicating a saturation-dependent component of the measurement error. $A_{\mathrm{rms}}$ should therefore be interpreted together with the limits of agreement, the distribution of individual measurement errors, and their dependence on saturation, rather than as the error expected for each individual measurement.

This saturation dependence was most pronounced in the 70–80% range, where the mean bias reached −2.44%, and warrants particular consideration because measurement errors at low oxygen saturation can be clinically relevant. Although this underestimation would be unlikely to obscure the presence of hypoxemia, it could exaggerate its apparent severity and potentially influence threshold-based clinical decisions or confirmatory testing [46]. Because the 70–80% range is already well below commonly used oxygen-saturation targets [47], the observed negative bias would be unlikely to change the recognition of marked hypoxemia itself, although it may affect assessment of its severity. The reduced accuracy at low saturation is consistent with previous controlled-desaturation studies reporting greater bias or error dispersion below 80% oxygen saturation [48,49]. Further evaluation in patients with naturally occurring hypoxemia is therefore needed to determine the clinical impact of this saturation-dependent bias.

Taken together, these findings support the technical feasibility of chest reflectance PPG as an optical signal source for SpO_2_ estimation under controlled desaturation conditions when combined with appropriate signal quality assessment and beat-wise analysis. Notably, the proposed algorithm was not limited to offline analysis but was implemented in the HiCardi M350 and evaluated by direct comparison with arterial SaO_2_.

### 4.1. Technical Feasibility and Potential Clinical Implications

The principal implication of this study is the technical feasibility of integrating ECG and SpO_2_ monitoring using a single chest-worn device. Conventional pulse oximeters are commonly placed on the finger or earlobe, where relatively strong PPG signals can be obtained. However, prolonged monitoring using a finger probe may be limited by wearability and signal stability during physical activity. Relative movement between the sensor and the finger can introduce measurement errors, and motion artifact is a major limitation of continuous SpO_2_ monitoring [50,51]. In a study of patients with chronic obstructive pulmonary disease using a wearable finger pulse oximeter, the proportion of valid SpO_2_ data decreased during moderate-to-vigorous physical activity because of motion artifact [13]. In addition, device comfort and user preference may influence device selection and long-term adherence in SpO_2_ self-monitoring [52].

The chest is suitable for patch-based monitoring and is spatially compatible with conventional ECG acquisition. A single chest patch can therefore support long-term acquisition of ECG, heart rate, respiration-related signals, and motion data. Previous studies have demonstrated the feasibility of continuous ECG-based heart rate and respiratory rate monitoring using chest-worn devices [53,54]. The clinical potential of chest-patch vital sign monitoring has also been investigated under motion and controlled hypoxia conditions [14]. Furthermore, sternum-based reflectance PPG has been proposed as a potential measurement configuration for wearable pulse oximetry [15]. Collectively, these findings suggest that chest-worn patches may provide a suitable platform for future integration of ECG and PPG-derived SpO_2_ measurements, although their performance during unrestricted daily activity requires further evaluation.

Integrated continuous monitoring may be particularly useful in settings that require prolonged physiological surveillance, including general wards, perioperative care, and post-discharge monitoring. A systematic review of continuous vital sign monitoring using wearable wireless devices in hospitalized patients suggested that continuous monitoring may facilitate earlier detection of clinical deterioration than conventional intermittent measurements [55]. The feasibility of continuous vital sign monitoring in the home after hospital discharge has also been reported [56]. In addition, wearable measurements of heart rate, respiratory rate, SpO_2_, and body temperature have been evaluated in patients recovering from surgery, supporting their potential use in perioperative and postoperative care [57]. The chest-based SpO_2_ estimation algorithm developed in this study provides a technical basis for incorporating oxygen saturation into such multimodal continuous monitoring platforms. However, the accuracy demonstrated in the present study was limited to controlled desaturation conditions and does not establish clinical performance during prolonged ambulatory or free-living monitoring or in patients with cardiopulmonary disease.

### 4.2. Algorithmic Considerations for Chest Reflectance PPG

Reliable SpO_2_ estimation from chest reflectance PPG requires stable extraction of the relatively small and variable pulsatile components of the red and IR PPG signals under low-perfusion conditions. Conventional pulse oximetry typically calculates the AC/DC ratios by detecting peaks and valleys in the red and IR PPG waveforms [4,5]. At reflectance measurement sites such as the chest, however, low perfusion, respiratory motion, and changes in sensor contact can destabilize peak–valley detection in the red and IR signals [15,16]. Therefore, rather than detecting beat landmarks directly from the red and IR waveforms, the proposed algorithm used green PPG, which generally exhibits a more distinct pulsatile component, as the reference signal for beat segmentation [27,29]. The detected green PPG beat timing was used to define HR-adaptive analysis windows, thereby reducing beat misalignment during changes in heart rate and ensuring that the corresponding red and IR PPG segments were included within each cardiac cycle.

The red-to-IR AC amplitude ratio was estimated using a least-squares-based linear transformation that did not depend directly on individual peak and valley locations. Conventional ratio-of-ratios estimation assumes that the AC component at each wavelength can be extracted reliably [15,16,17]. Under low-perfusion or motion-contaminated conditions, however, peak distortion, valley drift, and baseline fluctuation may increase the variability of peak-to-valley-based AC estimates [58,59]. The proposed approach therefore used all samples within each analysis window to regressively estimate the relative amplitude relationship between the red and IR PPG waveforms. A similar linear transformation between red and IR PPG beats was previously proposed for sternum-based reflectance pulse oximetry to overcome the limitations of peak and valley extraction in respiration-contaminated PPG [15]. Accordingly, the least-squares approach used in the present study was intended to reduce sensitivity to local waveform extrema and provide a more stable estimate of the *R* in chest PPG.

Signal quality was assessed using an NCC-based SQI. Correlation and template-matching-based SQIs have been used to evaluate the preservation of PPG beat morphology and exclude artifact-contaminated beats [15,60,61]. In the proposed algorithm, the green PPG beat was used as the reference waveform, and its morphological similarity to the corresponding red and IR PPG beats was assessed. Only beats satisfying the SQI criterion were included in the SpO_2_ calculation. Because motion artifact may cause transient false desaturation or isolated spikes [23,50], the output was not updated during low-SQI periods; instead, the most recent valid SpO_2_ value was retained. Median and moving-average filtering were then applied sequentially to suppress isolated spikes and physiologically implausible abrupt changes. Conversely, when SpO_2_ persistently decreased into the hypoxemic range, a dual-track strategy reduced the filter length to improve responsiveness. This framework was designed to balance output stability under normal conditions with the ability to track clinically important desaturation events and represents a key component of the chest-worn SpO_2_ estimation pipeline.

### 4.3. Comparison with Prior Work

The present study extends previous work on chest reflectance PPG-based SpO_2_ estimation and pulse oximetry signal processing. Chan et al. measured ECG and multiwavelength PPG using a wearable patch biosensor placed over the sternum and demonstrated the feasibility of sternum-based reflectance pulse oximetry using a breath-hold protocol [15]. To address unstable peak and valley extraction caused by low perfusion and respiratory artifact, their method incorporated green PPG-based outlier rejection and a least-squares linear transformation between red and IR PPG beats to derive the *R*. The present study shares the general algorithmic principles of green PPG-based beat-level quality assessment and least-squares estimation of the AC amplitude ratio.

However, the present study differs in both the reference measurement and the implementation of the algorithm. Chan et al. [15] evaluated sternum-derived SpO_2_ against finger pulse oximeter SpO_2_, whereas the present study used SaO_2_ measured using an arterial blood gas analyzer during controlled desaturation. Because the chest and finger may differ in their temporal responses and hemodynamic characteristics, direct comparison with arterial SaO_2_ reduces the contribution of site-dependent differences and provides a more direct assessment of algorithm accuracy. In addition, the analysis by Chan et al. was primarily performed offline and included breath-hold interval selection, alignment between finger SpO_2_ and chest PPG, and comparison of calibration schemes. In contrast, the present study integrated green PPG-based beat detection, ECG-assisted validation, HR-adaptive analysis windows, an NCC-based SQI, accelerometer-based motion artifact detection, and real-time post-processing into a continuous pipeline implemented on a chest-worn device.

Alkhoury et al. compared signal-processing strategies for mitigating motion artifact in pulse oximetry [59]. Their approach used comb filtering tuned to ECG-derived heart rate to preserve the cardiac fundamental and harmonic components of the PPG signal while attenuating motion-related noise between these frequencies. The present study similarly used ECG as an auxiliary signal for PPG-based SpO_2_ estimation. However, rather than using ECG-derived heart rate to tune a frequency-domain filter, it was used to assess the physiological plausibility of green PPG-derived pulse intervals and to define HR-adaptive analysis windows. Thus, whereas the approach of Alkhoury et al. focused on frequency-domain purification of motion-contaminated PPG, the present study implemented an integrated real-time pipeline for SpO_2_ estimation from low-perfusion chest reflectance PPG and evaluated its accuracy against arterial SaO_2_.

The principal contribution of this study is therefore the demonstration of the technical feasibility of real-time SpO_2_ estimation from chest reflectance PPG together with an evaluation of its accuracy against arterial SaO_2_ under controlled desaturation conditions. The proposed method provides a practical framework for incorporating SpO_2_ estimation into multimodal chest-worn monitoring systems.

### 4.4. Limitations and Future Directions

This study has several limitations. First, the number of participants used for algorithm calibration and accuracy evaluation was limited, and all 17 participants enrolled across the two study stages were young, healthy adults evaluated under controlled desaturation conditions. Accordingly, the performance of the proposed algorithm in older adults and in individuals with impaired circulation or low perfusion remains unknown and requires further evaluation. In addition, the subject-level LOSO analysis performed within the five-participant calibration cohort should be regarded as preliminary evidence of model robustness and generalizability and cannot substitute for independent external validation in a larger cohort. Although the Stage 2 accuracy cohort was independent of the Stage 1 calibration-development cohort, both were evaluated within the same overall clinical investigation. Therefore, the reported accuracy represents an independent within-study evaluation and should not be interpreted as external validation of algorithm performance.

More broadly, the study population did not adequately represent the full range of skin pigmentation, body habitus, chest tissue characteristics, and perfusion conditions that may be encountered in clinical and wearable settings. These factors may influence optical absorption and scattering, optical path length, pulsatile signal amplitude, and PPG signal quality and therefore may affect SpO_2_ estimation performance. Skin-pigmentation-related bias is particularly relevant to pulse oximetry; previous studies have reported greater SpO_2_–SaO_2_ errors, overestimation during hypoxemia, and a higher prevalence of occult hypoxemia in individuals with darker skin pigmentation [62,63,64]. Because standardized pigmentation measures such as the individual typology angle (ITA) or Monk Skin Tone (MST) were not prospectively collected and the present cohort was too small for reliable subgroup comparisons, pigmentation-stratified analysis was not performed. Future validation studies should therefore include larger and more diverse populations, including older adults and individuals with impaired circulation or low perfusion, prospectively characterize skin pigmentation, and evaluate bias, $A_{\mathrm{rms}}$, limits of agreement, and error distributions across pigmentation groups, with additional analyses according to body habitus, chest tissue characteristics, and perfusion status where sufficient sample sizes are available.

Second, the study was conducted under controlled desaturation conditions with restricted movement; therefore, performance in the presence of substantial motion artifacts encountered in daily life was not fully evaluated. Accordingly, the present results should not be interpreted as validation of performance during unrestricted or free-living continuous monitoring. Walking, postural changes, changes in sleeping position, and variations in sensor contact pressure may substantially distort chest PPG waveforms. Although green PPG provided a relatively stable reference for beat detection, SpO_2_ estimation ultimately depends on reliable extraction of the pulsatile AC components from the red and IR PPG signals. Therefore, when the red or IR pulsatile components are markedly attenuated, particularly under low-perfusion conditions, the reliability of *R* estimation and subsequent SpO_2_ estimation may be compromised even when green PPG-based beat detection remains stable. In addition, the NCC-based SQI may misclassify common-mode artifacts as acceptable beats when the green, red, and IR PPG signals are distorted in a similar manner. Future studies should evaluate the algorithm during ambulatory and prolonged wear and investigate a multimodal SQI framework that integrates accelerometer-derived motion indices, perfusion indices, PPG morphology, and beat-to-beat consistency. Separate performance testing under motion and low-perfusion conditions will also be necessary for devices intended for continuous monitoring.

Third, the magnitude and direction of the estimation error varied with oxygen saturation. The overall mean bias and $A_{\mathrm{rms}}$ were −0.70% and 2.91%, respectively, whereas the corresponding values in the nominal 70–80% range were −2.44% and 3.42%. The greater negative bias and wider dispersion observed at lower saturation levels warrant particular caution when interpreting performance in clinically significant hypoxemia. Moreover, although the nominal 70–80% range was represented in the dataset, observations near the lower boundary of the achieved SaO_2_ range were limited, restricting the strength of conclusions regarding performance at the most severe levels of desaturation. A significant proportional bias was also observed between the SpO_2_–SaO_2_ difference and mean saturation (slope =0.20, p<0.001), indicating increasing underestimation at lower saturation levels. As discussed above, this saturation-dependent underestimation may affect interpretation of hypoxemia severity and therefore represents an important limitation of the current calibration. Reduced perfusion, respiratory baseline fluctuations, changes in sensor contact pressure, and variations in tissue optical path length may exert a greater influence on *R* under hypoxemic conditions. Although the proposed method incorporated nonlinear calibration and multiple signal-quality control steps, a single global calibration equation may not fully capture the *R*–SaO_2_ relationship across the entire saturation range, particularly at low saturation levels. Future studies should include more observations in the hypoxemic range and evaluate saturation-dependent or physiologically constrained calibration approaches in independent clinical datasets while maintaining computational feasibility for real-time implementation.

Fourth, the post-processing framework, which included SQI freezing, median filtering, moving-average filtering, and dual-track filtering, involves an inherent trade-off between output stability and temporal responsiveness. Stronger smoothing can suppress artifact-related fluctuations but may delay the detection of true desaturation or resaturation, whereas greater responsiveness may increase the influence of transient artifacts on the reported SpO_2_ value. In particular, the SQI-freezing rule imposed no maximum holding duration in the current implementation; therefore, the most recent valid SpO_2_ value could remain displayed until a subsequent estimate satisfied the SQI criterion. During prolonged signal-quality degradation, this could result in an outdated SpO_2_ value remaining displayed. Future implementations should incorporate a bounded holding interval after which the output is invalidated. In addition, residual temporal mismatch may remain despite nearest-time pairing because physiological circulatory delay can vary among individuals, particularly during rapid desaturation or resaturation. This may increase the apparent bias and dispersion of the SpO_2_–SaO_2_ differences. Future studies should optimize post-processing parameters across a range of desaturation and resaturation rates and realistic wearing conditions, while also evaluating the sensitivity of accuracy estimates to interindividual variability in circulatory delay. Adaptive post-processing methods that dynamically adjust filter characteristics according to signal quality, rate of change, and current saturation level should also be investigated. Desaturation-event detection delay, recovery time, and false-alarm rate should be quantitatively assessed.

Finally, the present study was designed according to ISO 80601-2-61:2017, which was the applicable edition at the time of study design. Accordingly, the reported 4.0% $A_{\mathrm{rms}}$ criterion should be interpreted as the prespecified accuracy criterion used for the present study rather than as evidence of conformity with subsequently updated requirements. ISO 80601-2-61:2017 has since been superseded by ISO 80601-2-61:2026 [65]. In parallel, current regulatory recommendations have placed greater emphasis on evaluating pulse-oximeter performance across diverse skin pigmentation. In particular, the 2025 FDA draft guidance recommends standardized characterization of skin pigmentation using MST and ITA, broader representation across pigmentation groups, and reporting of accuracy and associated uncertainty both overall and across pigmentation strata [66]. Because the present study was not designed according to these updated standards and recommendations, its results should not be interpreted as demonstrating conformity with ISO 80601-2-61:2026 or with the proposed FDA performance framework. Future validation studies should therefore reflect the updated ISO standard and applicable regulatory recommendations.

In summary, the integration of green PPG-based beat segmentation, SQI-guided beat selection, least-squares estimation of the AC amplitude ratio, and real-time post-processing supported the technical feasibility of chest reflectance PPG-based SpO_2_ estimation and demonstrated its accuracy against arterial SaO_2_ under controlled desaturation conditions. Nevertheless, because the study involved a limited number of participants and was conducted under controlled desaturation conditions with restricted movement, the generalizability of the algorithm should be established in larger and more diverse populations, ambulatory and prolonged monitoring settings, datasets containing sufficient observations in the hypoxemic range, and independent external validation cohorts. Further improvement in low-saturation calibration and dynamic response, together with validation against updated standards and applicable regulatory recommendations, may facilitate the extension of this algorithm to continuous wearable monitoring systems that simultaneously provide ECG and SpO_2_.

## 5. Conclusions

In this study, a real-time SpO_2_ estimation algorithm integrating green PPG-based beat segmentation, signal quality assessment, least-squares estimation of the AC amplitude ratio, and adaptive post-processing was developed and implemented in the HiCardi M350 chest-worn device. When evaluated against arterial SaO_2_ during a controlled desaturation study, the algorithm achieved a pooled $A_{\mathrm{rms}}$ of 2.91% over the reference SaO_2_ range of 73–97%, satisfying the prespecified accuracy criterion of 4.0%. These findings support the technical feasibility of chest reflectance PPG-based SpO_2_ estimation and demonstrate its accuracy under controlled desaturation conditions when combined with appropriate signal quality control and beat-wise analysis. The proposed approach may provide a basis for future development of multimodal chest-worn monitoring systems capable of providing simultaneous ECG and SpO_2_ measurements. However, because the present study was conducted in healthy volunteers under controlled desaturation and restricted-movement conditions, further validation in larger and more diverse populations, including patients with cardiopulmonary disease, and under ambulatory, prolonged-wear, and free-living conditions is required to establish its applicability to continuous real-world SpO_2_ monitoring.

## Figures and Tables

**Table 1:** Agreement between the M350 pulse oximeter and the reference arterial oxygen saturation, stratified by reference saturation range. Each column summarizes all pairs falling within that range, pooled across the 12 participants; the final column covers the full range after pooled-data trimming. Confidence intervals were obtained by participant-level (cluster) bootstrap resampling with 10,000 replicates, which accounts for the repeated measurements contributed by each participant.

Metric	Reference SaO_2_ Range (%)			
	70–80 (n=91)	80–90 (n=92)	90–100 (n=95)	70–100 (n=278)
Mean bias (%)	−2.44	−0.44	0.71	−0.70
95% CI (%)	−3.35 to −1.54	−1.59 to 0.83	−0.12 to 1.60	−1.58 to 0.15
Standard deviation (%)	2.41	3.03	2.02	2.83
Maximum bias (%)	1.30	6.50	6.00	6.50
Minimum bias (%)	−8.40	−8.00	−5.90	−8.40
Precision (%)	2.46	2.99	2.19	2.54
Between-subject variance of bias ($\sigma_{u}^{2}$)	2.59	3.69	1.74	2.39
Within-subject variance of bias ($\sigma^{2}$)	3.51	5.58	2.29	5.81
Intraclass correlation coefficient	0.43	0.40	0.43	0.29
Excluded by data trimming (*n*)	0	0	10	10
$A_{\mathrm{rms}}$ (%)	3.42	3.04	2.13	2.91
95% CI (%)	2.51 to 4.17	2.35 to 3.64	1.64 to 2.66	2.45 to 3.34

**Table 2:** Agreement between the M350 pulse oximeter and the reference arterial oxygen saturation, stratified by participant. Each row summarizes the paired samples contributed by one participant after data trimming. The final two rows report the mean and standard deviation of these values across the 12 participants.

Participant	*n*	Reference SaO_2_ (%)		Bias	SD	$A_{\mathrm{rms}}$	MAE	Pairs Within
		Min.	Max.	(%)	(%)	(%)	(%)	±3% (% of *n*)
1	24	75.1	95.5	+0.66	2.12	2.17	1.76	91.7
2	24	77.1	96.8	−0.15	2.27	2.23	1.88	87.5
3	24	76.1	93.4	−2.18	2.98	3.65	3.22	58.3
4	24	74.0	94.3	+1.87	2.26	2.89	2.24	70.8
5	21	75.9	97.0	−2.86	1.83	3.37	2.90	47.6
6	24	76.1	96.1	−0.77	2.07	2.17	1.47	83.3
7	21	73.5	96.5	−1.32	2.77	3.01	2.45	81.0
8	24	74.0	94.6	−2.70	3.39	4.28	3.27	58.3
9	23	75.4	97.0	−0.59	2.30	2.33	1.76	78.3
10	21	75.3	96.6	+1.61	2.28	2.75	2.00	71.4
11	24	73.1	95.6	+0.33	1.35	1.37	1.10	95.8
12	24	73.8	96.6	−2.34	2.60	3.46	2.49	70.8
Mean across participants (n=12)		–	–	−0.70	2.35	2.81	2.21	74.6
SD across participants (n=12)		–	–	1.63	0.53	0.80	0.68	14.6

## Data Availability

The data presented in this study are available on request from the corresponding author. The data are not publicly available due to privacy restrictions applicable to clinical investigation data.

## Abbreviations

The following abbreviations are used in this manuscript:

AC	Alternating current (pulsatile) component
ACC	Accelerometer
MAE	Mean absolute error
ADT	Adaptive threshold detection
$A_{\mathrm{rms}}$	Accuracy root mean square
DC	Direct current component
ECG	Electrocardiogram
FIR	Finite impulse response
HR	Heart rate
IR	Infrared
ITA	Individual typology angle
LOSO	Leave-one-subject-out
MST	Monk Skin Tone
$S_{\mathrm{res}}$	Residual standard error
NCC	Normalized cross-correlation
PPG	Photoplethysmography
*R*	Ratio of ratios
RMSE	Root-mean-square error
SaO_2_	Arterial oxygen saturation
SpO_2_	Peripheral oxygen saturation
SQI	Signal quality index
LoA	Limits of agreement
